# Therapeutic Potential of Naringenin Nanosuspension: In Vitro and In Vivo Anti-Osteoporotic Studies

**DOI:** 10.3390/pharmaceutics14071449

**Published:** 2022-07-11

**Authors:** Sonia Gera, Sunitha Sampathi, Sravya Maddukuri, Sujatha Dodoala, Vijayabhaskarreddy Junnuthula, Sathish Dyawanapelly

**Affiliations:** 1 Department of Pharmaceutics, National Institute of Pharmaceutical Education and Research (NIPER), Hyderabad 500037, India; sonia.gera060@gmail.com; 2 GITAM School of Pharmacy, GITAM Deemed to Be University, Rudraram, Hyderabad 502329, India; smadduku@gitam.edu; 3 Institute of Pharmaceutical Technology, Sri Padmavati Mahila Visvavidyalayam, Tirupati 517502, India; drsujathasai@gmail.com; 4 Drug Research Program, Faculty of Pharmacy, University of Helsinki, Viikinkaari 5 E, 00790 Helsinki, Finland; 5 Department of Pharmaceutical Science and Technology, Institute of Chemical Technology, Mumbai 400019, India

**Keywords:** naringenin, nanosuspension, ovariectomized, osteocalcin, osteoporosis, bone, nanomedicine

## Abstract

Naringenin (NRG) is a flavonoid and has been reported as an anti-osteoporotic agent. However, poor bioavailability may limit the anti-osteoporotic potential of the drug. The purpose of the study was to compare the anti-osteoporotic activity of naringenin nanosuspension (NRG-NS) with the NRG and standard therapeutic drug, raloxifene hydrochloride (RLX). Here, NRG-NS showed anti-osteoporotic activity in MG-63 cells by upregulating the osteocalcin levels. The in vivo anti-osteoporotic activity of NRG-NS was further investigated in an osteoporotic rat model to mimic the post-menopausal condition. The animals were randomized and separated into six groups. The animals were treated with RLX (p.o., 5.4 mg/kg), NRG (p.o., 20 mg/kg), NRG-NS (p.o., 20 mg/kg), and blank-NS for 60 days after completion of a 30-day post-surgery period and compared with control and ovariectomized (OVX) groups. After the treatment, body and uterine weights, biochemical estimation in serum (calcium, phosphorus, acid phosphatase, alkaline phosphatase, osteocalcin), bone parameters (length, diameter, dry weight, density, ash weight, bone mineral content) and bone microarchitecture by histopathology were determined. The results showed the protective effects of NRG-NS on osteoblast-like MG-63 cells. The biochemical estimations confirmed the normalization of parameters viz., alkaline phosphatase, calcium concentrations, and bone density with a decrease in levels of acid phosphatase and inorganic phosphorus with NRG-NS as compared to plain NRG. The results indicated that the oral administration of NRG-NS could be a potential therapeutic formulation for the treatment of osteoporosis.

## 1. Introduction

Osteoporosis (OP) is a bone disorder characterized by impaired bone mineral density (BMD), decreased bone strength, and micro-architecture deterioration leading to the risk of fractures [1]. This disease harms patients’ quality of life, resulting in substantial medical and socioeconomic effects, in particular for women above 40 years of age [2,3]. Osteoporotic fractures are the major cause of morbidity and mortality, especially in developed countries, accounting for 0.83% of the global burden among non-communicable diseases [4]. The pharmacological therapies used to reduce the risk of fractures are categorized as anti-resorptive agents such as estrogen, bisphosphonates, selective estrogen receptor modulators (SERMs), calcitonin, and vitamin D analogs, the combination of calcium and vitamin D, and anabolic agents i.e., teriparatide and sodium fluoride [5]. Even though medications are effective, most of them are associated with significant side effects with long-term administration [6]. Some of the side effects are osteonecrosis of the jaw, esophageal disturbance, hypocalcemia, nausea, diarrhea and thromboembolism [3]. Estrogen is a hormone playing a dominant role in controlling the typical bone homeostasis. Estrogen deficiency leads to bone loss in postmenopausal osteoporosis (PMO) [7]. Long-term estrogen therapy in women has been linked to breast, endometrial, and ovarian cancer, as well as venous thromboembolism and stroke, according to epidemiological research [8]. Natural therapies have been found to enhance bone health and prevent bone loss by increasing bone mineral density (BMD) and protecting against antioxidant and anti-inflammatory stress [9,10]. Flavonoids are plant-derived secondary metabolites with a beneficial effect on health [11,12]. Despite the bioactivity shown in in vitro investigations, the poor bioavailability restricts the therapeutic activity [13]. 

Numerous nanomedicine approaches have been reported in the literature to improve the therapeutic potential and retention of drug molecules in various therapeutic indications [14,15,16,17,18,19,20,21]. Nanocarriers can considerably improve flavonoids’ deficiencies by increasing solubility and absorption, and avoiding degradation [13]. Naringenin (NRG) is a citrus flavonoid found in grapefruit that has been shown to help with osteoporosis, cancer, and cardiovascular disease owing to its antioxidant and anti-inflammatory properties [22]. Naringin (NG) has been reported to stimulate bone formation by promoting osteoblast differentiation and boosting bone robustness in ovariectomized animals [23,24,25]. Recent research supports naringenin’s therapeutic potential in osteoporosis [26]. However, its poor bioavailability (5%) limits its medicinal potential [27]. Nanosuspensions (NS) can improve the bioavailability of poorly soluble drugs by increasing the dissolution through the reduction in particle size, increased surface area and dissolution [28]. Other significant benefits of NS includes improvement in bioavailability, dose proportionality, reduction in fed/fasted variability, reduction in inter-subject and enhanced absorption rate [29,30]. The significant difference between other nanomedicines and nanosuspensions lies in the fact that they do not need a carrier portion, hence the maximum drug loading is possible. In some instances, nanosuspensions have been prepared without any additives, which implies 100% drug availability for therapeutic dosage [31]. MK Singh et al. developed a stable NRG nanosuspension (NRG-NS) by the precipitation-ultrasonication method using different concentrations of surfactants and polymers. The resulted formulation displayed enhanced solubility and improved pharmacokinetic profile in male Sprague Dawley rats [32].

Our earlier research investigated formulation and pharmacokinetic aspects of NRG-NS and demonstrated enhanced bioavailability [33]. The current research aimed to study the activity of NRG-NS on in vitro osteoblast MG-63 cell lines and an in vivo osteoporotic rat model. 

## 2. Materials

Naringenin (MW = 272.25, purity > 95% purity), polyvinyl pyrrolidone (PVP K90), 3-(4,5-dimethylthiazol-2-yl)-2,5-diphenyltetrazolium bromide (MTT) and dichlorofluorescin diacetate (DCFDA), minimum essential medium (MEM), fetal bovine serum (FBS), non-essential amino acids, trypsin, ethylene diamine tetraacetic acid (EDTA), formalin, eosin stain, and hematoxylin were procured from Sigma-Aldrich (St. Louis, MO, USA). Human osteocalcin ELISA kit was purchased from Invitrogen (R &D systems, Minneapolis, MN, USA). Raloxifene hydrochloride was obtained from Dr. Reddy’s laboratories (Telangana, India). Ketamine and xylazine were bought from Indian Immunological Private Limited (Andhra Pradesh, India). Iodopovidone, ciprofloxacin, and neomycin antibiotic powder were purchased from Keimed (Hyderabad, India). Calcium, alkaline phosphatase phosphorus, and acid phosphatase kits were procured from Accurex Biomedical Pvt. Ltd. (Mumbai, India). The remaining reagents and solvents are of analytical grade. Surgical sutures (Lotus Surgical Pvt Ltd., Mumbai, India), and suture needles (Microsharp Needles, Bengaluru, India) were purchased from the standard suppliers.

### 2.1. Formulation of NS

NRG-NS formulation was prepared using the sonoprecipitation method as described earlier [33]. A total of 1 mL of organic phase (NRG in ethanol solution, 30 mg/mL) was injected immediately into 10 mL of aqueous PVP K-90 solution (anti-solvent system, 0.5% *w*/*v*) under magnetic stirring at 1000 rpm for 5 min and further processed by the ultrasonic processor (Sonics & Materials, Inc., Vibra cell VCX 750, Newtown, CT, USA). Later, freeze dried formulations were further used for subsequent studies. 

### 2.2. Cell Culture

The osteogenic sarcoma cell line (MG-63; human osteoblast-like) was procured from the American Type Cell Culture (ATCC^®;^ ID No: CRL-1427™, ATCC, Manassas, VA, USA). The cell lines were maintained in minimum essential medium (MEM) complemented with fetal bovine serum (FBS; 10%, Sigma-Aldrich (St. Louis, MO, USA), augmented with non-vital amino acids (1%) in a CO_2_ incubator (5%) at a relative humidity of 90%. After reaching confluency (80–90%), the cells were treated with trypsin:EDTA (0.25%:1 mM) solution and subjected to further passing. 

### 2.3. In Vitro Activity on MG-63 Cell Lines

#### 2.3.1. Cell Viability Assay

The cytotoxic evaluation of NRG, NRG-NS and blank-NS particles was determined by MTT assay. MG-63 (cell lines) of 1 × 10^4^ cells per well were grown in 96-well microculture plates in 100 µL MEM, supplemented with 10% FBS and incubated for 24 h at 37 °C in a CO_2_ incubator. After 24 h, the culture media was withdrawn, and each well was treated with 100 µL of NRG, NRG-NS, and blank-NS (concentration range 10–0.15 g/mL) and maintained for 48 h. The MTT reagent (0.5 mg/mL) of 100 µL was added to each well and further incubated for 48 h in the dark at 37 °C. The supernatant from each well was cautiously separated, formed formazan crystals were dispersed in 200 µL of DMSO and absorbance was determined at 570 nm.

#### 2.3.2. Determination of Osteocalcin Levels

To assess the osteocalcin level, the MG-63 cell lines at a seeding density of 1 × 10^4^ cells per well were maintained for 24 h and then incubated with NRG, NRG-NS, and blank-NS (concentration range 0.5 µg/mL) for 48 h. The culture supernatants were collected according to the manufacturer’s instructions included with the kit.

### 2.4. Animal Study

Adult female Wistar rats (200–220 g) were maintained in the institute’s animal house. Animals were kept under controlled temperature conditions (23 ± 2 °C) and humidity (50 ± 5 %). The rats were supplied with food and water as per the guidelines. The experimentation was accomplished according to the “Guidelines of the Committee for Control and Supervision of Experiments on Animals (CPCSEA)’’. The procedure was accepted by Institutional Animal Ethical Committee (IAEC) with protocol number NIPER/2016/PE/187.

#### 2.4.1. Anti-Osteoporotic Activity

After a week of adaptation, the rats underwent surgery. Ovariectomy was conducted under anesthesia (ketamine (50 mg/kg, i.p) and xylazine (5 mg/kg, i.p)). The ovaries’ location was approximately marked in the rat abdomen by carrying out two long dorso-lateral incisions of a length of 3 cm. The ovaries were then surgically removed, followed by applying ciprofloxacin drops (0.2 mL) to prevent any infection [34,35]. Suturing with sterile absorbable catguts was carried out immediately to stop bleeding. Stitches were applied on both the sides of the abdomen and the povidone-iodine solution was applied daily for a week. The ovariectomized rats were allowed to recover for 30 days from the date of surgery. Later, the treatment was carried out from the 31st day to the 90th day. After the completion of the treatment period (i.e., on the 91st day), the animals were sacrificed, and bones and uterus were collected for further investigations.

#### 2.4.2. Grouping of Animals and Assigning Treatment

The rats were randomly allocated into 6 sets of 8 rats each. Group I served as a sham group (control) in which the surgery was carried out. The ovaries were uncovered but not separated. In the other groups of animals, ovariectomy was performed to induce osteoporosis and further the animals were divided into groups II to VI. Normal saline was given to group II (OVX) during the treatment time (disease self-control group). In group III, standard drug RLX was administered (dose 5.4 mg/kg, p.o) [36]. NRG, NRG-NS and blank-NS were administered per oral route of 20 mg/kg body weight to groups IV, V and VI. After 30 days post-surgery, every morning the animals in the respective groups were given oral gavage of formulation and the treatment was continued for 2 months i.e., 90 days in total. The number of animals (n = 8) per group was approved by the IAEC; four animals per group were used for histopathological analysis and the remaining four animals were used for assay of bone density.

#### 2.4.3. Biochemical Estimation (Serum and Urine)

From all the groups of animals, the blood samples were drawn from the aorta and the serum was harvested by centrifugation (5000 rpm) and stored until further analysis of biochemical parameters. Animals of the respective group were then individually accommodated in metabolic enclosures for 24 h for collection of urine before sacrifice, during which the animal was supplied with unionized water. The collected samples (urine) were acidified using hydrochloric acid (0.03% *v/v*). Estimation of calcium and phosphorus levels in the respective samples (serum and urine) was determined using a colorimetric technique. Serum alkaline phosphatase (ALP) and acid phosphatase (ACP) concentrations were determined using an Accurex biochemical kit, Accurex Biomedical Pvt. Ltd., Mumbai, India.

#### 2.4.4. Bone Analysis

Femur bones were removed after sacrificing the female rats. The bone length, diameter and bone dry weight were assessed. A calibrated vernier calipers (Mitutoyo 531 series; Mitutoyo Corporation, Kanagawa, Japan) was used for assessing the bone length and diameter. The calcium and phosphorus content was determined by subjecting the femur bones to initial drying in an oven at 100 °C (12 h), followed by incineration at 1000 °C to generate ash [37]. Calcium and phosphorus content in the ash were determined by employing the colorimetric technique. Bone density was measured with Archimedes’ principle and calculated by the following Equation (1) [38].

(1) $$D=\left(\frac{W1}{W1-W2}\right)\text{×}P\;$$

where “D” is the density, W_1_ and W_2_ are the weight in air and water respectively and “P” is the density of water at a given temperature.

#### 2.4.5. Body and Uterine Weights

Before and after the start of the treatment (0th and 91st day), the body weights were taken for all the animals in the respective groups. Using CO_2_ euthanasia, rats were sacrificed after the treatment schedule and uterine horns were removed; these weights were noted. 

#### 2.4.6. Histopathology

The femur bones (right leg) from each group were stored in formalin (10%) for two days. Decalcification of the bones was done by placing in EDTA (5%) for seven days. The bone samples were then held in paraffin wax; using a microtome (Leica, Munich, Germany), a diagonal section along the sagittal plane passing via transverse alignment of 5µ thickness was taken. The sections were stained with hematoxylin and eosin and studied under an optical microscope for histopathological changes [39].

#### 2.4.7. Statistical Assessment

All the results are stated as the mean ± S.E.M; “n” refers to the number of samples studied. The statistical variations between the means (maximal relaxation) were verified by one-way ANOVA utilizing GraphPad Prism computer software (edition 6.01; Graph Pad, San Diego, CA, USA). *p* ≤ 0.05 was considered statistically significant.

## 3. Results and Discussion

Nanocarriers are a solution to provide better health benefits for bone strength in osteoporosis. Recent reports of nanocarriers in similar areas show encouraging results for dealing with bone loss. Ovariectomy-induced bone damage and post-menopausal bone damage share many comparable characteristics and rats represent the best animal model to study osteoporosis [40]. In the present study, the optimized and well-characterized NRG-NS formulation was evaluated for anti-osteoporotic activity. The characterization and pharmacokinetic parameters of NRG-NS were reported in our previous publication [33]. The NRG-NS used in the current study showed a particle size of 117 ± 5 nm and zeta potential of −14.6 ± 5.6 mV [33]. This is in consistency with previously reported NRG-NS with different polymeric surfactants; the zeta potential in this range was suitable for excellent stability as previously reported [32]. In the present study, the optimized and well-characterized NRG-NS was evaluated for in vitro and in vivo anti-osteoporosis activity. 

### 3.1. Cell Viability Assay

**Figure 1:**
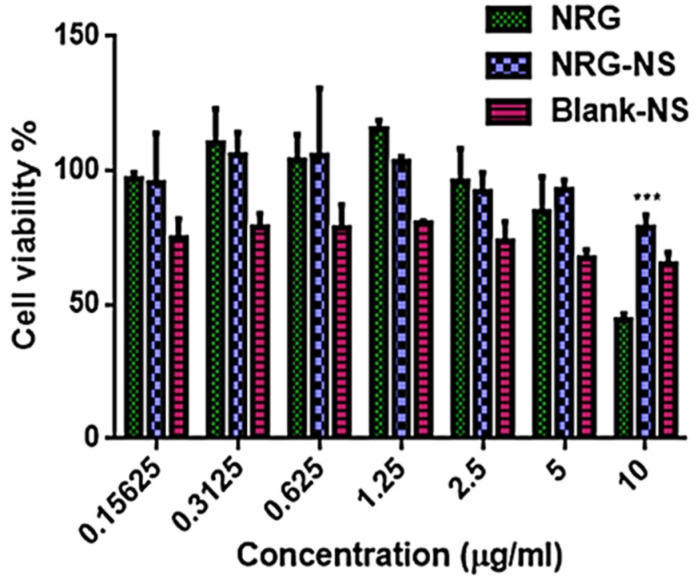
Cell viability assay on MG-63 cell lines. Cytoprotective result on cells (0.15–10 µg/mL) for 48 h. MTT incubation after treating with NRG-plain, NRG-NS and blank-NS. Mean ± S.E.M (n = 3). *** *p* < 0.001 is considerably diverse from the NRG plain.

The MG-63 cell viability was determined by employing an MTT assay. MG-63 cell lines have the advantages of hormonal administration response similar to human osteoblast and human integrin subunits profiles [41]. The cells were treated with different concentrations (0.15–10 µg/mL) of NRG, NRG-NS, and blank-NS. Results showed that there was no substantial change in the proliferation with the NRG-NS group as compared to the NRG group except at 10 µg/ml concentration (Figure 1). Nevertheless, no significant changes in cell viability with blank-NS were observed. Naringenin enhances the osteoblast expression by increasing the osteoprotegerin (OPG) levels and BMP expression in vitro, as reported earlier [42]. In the current study, NRG-NS were found to be safe at the concentrations studied and the dose of 0.5 µg/mL was selected for further studies.

### 3.2. Effect of Formulation on Levels of Osteocalcin in Cell Lines

**Figure 2:**
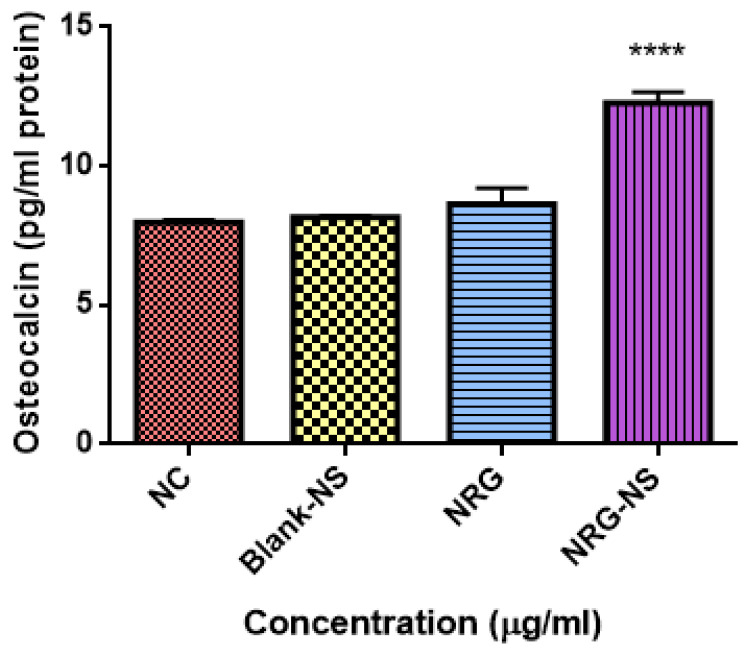
The concentration of osteocalcin secretion in MG-63 cells after treatment with NRG-plain, NRG-NS and blank-NS (0.5 µg/mL) for 48 h. The values presented are means ± S.E.M (n = 3). **** *p* < 0.0001 is significant. Note: NC: negative control.

Osteocalcin (OCN) is a bone protein secreted by osteoblasts during the bone formation in the bone remodeling process; it is an important diagnostic biomarker in osteoporosis [43]. The effect of NRG-NS was explored by ascertaining the levels of OCN synthesis in MG-63 cell lines. Because of previous reports, naringenin is superior to naringin in enhancing the osteocalcin levels [23]. NRG-NS significantly enhanced osteocalcin secretion at 0.5 µg/mL compared with standard control (Figure 2). 

### 3.3. Effect of Formulation on Body Weight and Uterine Weight

The impact of RLX, NRG, NRG-NS, and blank-NS in ovariectomized rats on body weight and uterine weight was examined (Table 1). These parameters can ensure the efficiency of the OVX procedure in female rats. A significant variation in body weights of animals in the OVX group was noticed compared to the sham group. However, no major change between the different treatment groups was observed during the study period. The weight gain in animals indicates a deficiency in estrogen levels. The reduction in estrogen levels may be due to fat loss under the skin and increased abdominal fat, as per prior literature [44]. Unlike body weight, the rat’s uterine weights reduced considerably in the OVX group (untreated rats) in comparison to the sham group, whereas the treatment groups had displayed values (uterine weight) almost equal to a regular control group; the results agreed with other reported studies [45]. Compared to the earlier studies reported in the literature, treatment with NRG leads to higher uterine weights than OVX and other groups [46]. The uterotrophic effects of NRG were reported due to interaction with the ER-α receptor [47]. Both NRG and NRG-NS produced an almost similar increase in the uterus weights except for blank-NS.

### 3.4. Biochemical Parameters (Serum and Urine)

**Figure 3:**
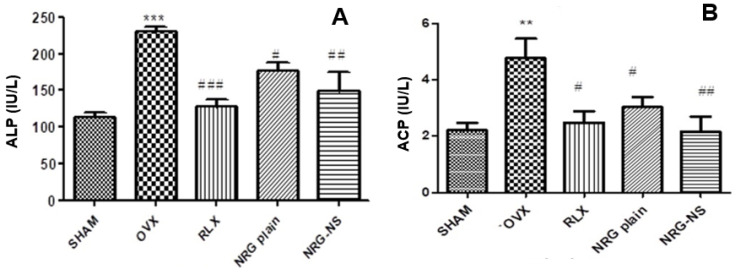
(**A**) ALP levels in serum of various animal groups. The values presented are mean ± S.E.M (n = 8). ALP *** *p* < 0.001 vs. sham, ^#^ *p* < 0.05, ^##^ *p* < 0.01, ^###^ *p* < 0.001 vs. OVX SHAM. (**B**) ACP levels in serum of various animal groups. ACP ** *p* < 0.01 vs. sham, ^#^ *p* < 0.05 ^##^ *p* < 0.01 vs. OVX control. OVX SHAM—sham control, OVX—ovariectomized control, RLX—plain raloxifene hydrochloride (as standard), NRG or NRG-plain, NRG-NS.

Deformation of typical bone structure and resorption are the critical factors responsible for osteoporosis [48]. The exaggerated rates of bone resorption are marked by a high level of acid phosphatase leading to osteoclast activation and bone resorption [49]. Simultaneously, increased serum OCN concentrations are positively associated with improved bone formation [50]. Hence, alkaline phosphatase and OCN concentrations related to bone formation and mineralization were assessed in our study. There was a noteworthy increase in serum levels of OCN and ALP with a substantial decrease in serum levels (calcium, phosphorus, and total protein content) in the ovariectomized rats, as compared to the sham control group, confirming the induction of osteoporosis. The different treatment groups ameliorated the altered parameters (Table 2) when treated with RLX (standard drug), NRG and NRG-NS. Raloxifene hydrochloride treatment can alter the mineral calcium and phosphate absorption and excretion, similar to previous reports [51]. The bone-enhancing property of NRG may be possibly related to the positive role of estrogen receptors leading to the differentiation of bone components [25]. The NRG-NS treated group enhanced the ALP levels more than NRG (Figure 3A), which can be credited with increased bioavailability of NRG when dispensed in NS form. This provides evidence of the critical role of flavanone relating to its higher biological efficacy [52]. Acid phosphatase activity precisely reflects the action of osteoclasts. Our findings also support a significant escalation in osteoclastic activity, suggesting higher resorption of bone in the ovariectomized rats (Figure 3B). 

RLX-treated standard group resulted in increasing acid phosphatase levels and thereby represented a decrease in the elevated bone resorption activity. NRG-NS was able to control these levels more effectively than NRG and blank-NS. The current investigation demonstrated that OVX rats displayed higher concentrations of osteocalcin and were responsible for osteoclastic activity concerning lower bone turnover. At the same time, the treatment group also showed higher OCN levels. The increased serum OCN levels were most likely due to newly synthesized protein, as well as that released from the bone matrix during resorption as per the earlier reports [53]. Further studies are necessary to investigate the exact reason for enhanced OCN levels with NRG and NRG-NS. 

Under the femur physical parameters, bone length and diameter did not show considerable change (Table 3). The bone parameters such as bone dry weight and density were reduced appreciably in OVX grouping as contrasted to the control (*p* < 0.05). RLX-, NRG- and NRG-NS treated groups resulted in restoring the bone mass near to normal. A substantial increase in urine calcium and phosphorus levels was noticed in OVX control rats compared with the sham control group. In the case of bone ash, the calcium levels were significantly decreased. However, no considerable difference was observed in the phosphorus levels in the OVX group in contrast to the sham control group, indicating bone demineralization, whereas in the treated groups, the levels (calcium and phosphorous) were normalized with various treatments (RLX, NRG, and NRG NS) except for the group treated with the blank-NS. These results suggest that the anti-osteoporotic effect of NRG-NS is comparatively better than NRG.

### 3.5. Histopathology

**Figure 4:**
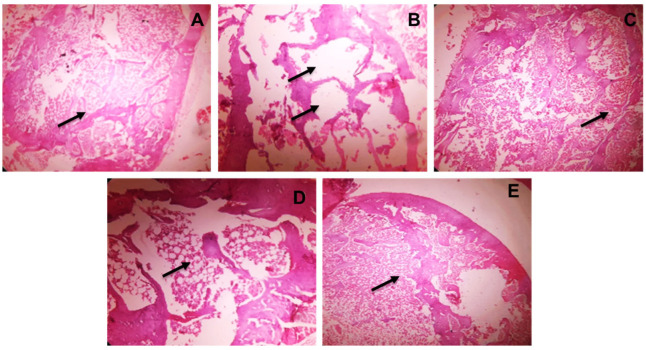
(**A**–**E**) Histopathological images of rat bone at the area of the tibial joint (10× magnification). (**A**) Sham control displaying regular trabecular construction (Indicated by arrows). (**B**) OVX control displaying the damage of interconnectivity, diminishing trabecular construction and lytic variations (Indicated by arrows). (**C**) Raloxifene group shows rebuilding of normal trabecular construction of bones (Indicated by arrows). (**D**) Naringenin plain also causes restoration of trabecular spaces (Indicated by arrows). (**E**) NRG-NS shows bone renewal (Indicated by arrows).

Osteopenia is found in OVX rats within two weeks of post ovariectomy. The condition was gradually improved (90 days), as evidenced by histological alterations in terms of trabecular bone degradation and micro-architecture weakening due to estrogen insufficiency [54]. Treatment with RLX, NRG, and NRG-NS showed repaired architecture of cortical and trabecular bone. A well-organized bone matrix was also discovered, which was linked to increased fracture resistance (Figure 4).

## 4. Conclusions

The relevance of nutritional components and food in minimizing the bone loss caused by estrogen shortage in OP has recently received a lot of attention. The current study aimed to investigate the effects of NRG-NS in an ovariectomized rat model. The in vivo results indicated increased bone density with NS of NRG compared to plain NRG, while there were only minor variations in bone mineral levels of calcium and phosphorus in serum and urine after treatment. More research could be carried out employing a similar technique for bioactive molecules with low bioavailability in this area. The study can be concluded by highlighting that enhancing the bioavailability of naringenin can improve the in vitro and in vivo bone formation activity and thus provide a better solution in the treatment of osteoporosis. Further strategies need to be devised to establish the relationship of serum OCN levels concerning bone formation or resorption. 

## Figures and Tables

**Table 1:** Effect of treatment on body weight and uterine weight.

S. No.	Parameters	Initial Weight (g)	Final Weight (g)	Weight Variation (g)	Uterine Weight (g)
1	Sham	189.12 ± 8.22	205.32 ± 12.54	16.2 ± 4.32	0.56 ± 0.15
2	OVX (Control)	190.45 ± 9.65	284.19 ± 23.32 ***	93.74 ± 3.67	0.09 ± 0.02 ***
3	RLX	205.12 ± 5.43	257.86 ± 18.98	52.74 ± 3.55	0.16 ± 0.02 ^###^
4	NRG	216.23 ± 2.13	240.74 ± 28.90	24.51 ± 6.77	0.13 ± 0.07 ^#^
5	NRG-NS	209.15 ± 1.98	225.52 ± 17.87	16.37 ± 5.89	0.15 ± 0.06 ^##^
6	Blank-NS	192.64 ± 5.87	228.81 ± 18.76	36.17 ± 2.89	0.10 ± 0.03

The values presented are mean ± S.E.M (n = 8); *** *p* < 0.001 vs. sham, ^#^ *p* < 0.05, ^##^ *p* < 0.01, ^###^ *p* < 0.001 vs. OVX, SHAM: sham, OVX: ovariectomized (control), RLX: raloxifene, NRG: naringenin, NRG-NS: naringenin nanosuspension.

**Table 2:** Effects of different treatments on biochemical estimations (serum and urine).

S. No.	Treatment	Calcium Estimation in Urine (mg/dL)	Serum Calcium Estimation (mg/dL)	Serum Phosphorus Estimation (mg/dL)	Total Protein (g%)	Phosphorus Estimation in Urine (mg/dL)	Serum OCN (ng/mL)
1	Sham	5.02 ± 2.78	10.05 ± 1.06	25.53 ± 0.52	10.05 ± 1.40	4.75 ± 1.98 *	2.20 ± 1.7
2	OVX untreated	4.45 ± 0.38	5.90 ± 0.47 ^###^	22.31 ± 0.09	9.98 ± 0.87	2.29 ± 0.95	2.53 ± 1.90 **
3	RLX	4.55 ± 0.82	9.10 ± 0.25 **	27.95 ± 1.42	10.13 ± 1.12	6.38 ± 2.77 *	2.19 ± 1.25 *
4	NRG	4.91 ± 0.72	7.63 ± 1.19 *	25.89 ± 0.23	9.57 ± 1.20	3.96 ± 0.81	2.47± 1.98 **
5	NRG-NS	3.81 ± 0.99	9.83 ± 0.87 **	26.31 ± 0.48	9.56 ± 2.76	4.18 ± 0.70	3.89 ± 1.5 ^##^
6	Blank-NS	4.23 ± 0.34	6.34 ± 0.04	21.76 ± 0.34	9.06 ± 1.43	2.65 ± 0.23	2.32 ± 1.54

The values presented are mean ± S.E.M (n = 8); Note: RLX: raloxifene, NRG: naringenin, NRG-NS: naringenin formulation, OVX: ovariectomized * *p* < 0.05 vs. OVX untreated group, * *p* < 0.05 vs. sham; ^##^ *p* < 0.01, ^###^ *p* < 0.001, ** *p* < 0.01, * *p* < 0.05, RLX, NRG, NRG-NS and Blank-NS vs. OVX untreated.

**Table 3:** Bone parameters in various groups after formulation treatment.

S. No.	Group	Weight (g)	Ash Weight (g)	Length (mm)	Diameter (mm)	Bone Density (g/cm^3^)	Bone Inorganic Content (mg%)	
							Ca	P
1	SHAM	0.88 ± 0.18	0.07 ± 0.02	28.46 ± 1.28	2.48 ± 0.32	1.79 ± 0.008	10.27 ± 0.40	4.19 ± 0.08
2	OVX Control	0.58 ± 0.07 ^#^	0.03 ± 0.01 ^#^	27.76 ± 0.30	2.09 ± 0.05	0.54 ± 0.09 ^##^	5.25 ± 0.65 ^##^	3.76 ± 0.05 ^#^
3	RLX	0.80 ± 0.08	0.08 ± 0.02	28.01 ± 0.49	2.17 ± 0.01	1.80 ± 0.07 **	11.57 ± 2.19	4.64 ± 0.01
4	NRG	0.70 ± 0.07	0.08 ± 0.01	26.66 ± 1.24	2.54 ± 0.34	0.69 ± 0.07	11.48 ± 1.45	4.20 ± 0.04
5	NRG-NS	0.85 ± 0.12	0.08 ± 0.01	27.86 ± 1.13	2.56 ± 0.76	1.40 ± 0.29 *	11.09 ± 0.05	4.06 ± 0.03
6	Blank-NS	0.61 ± 0.11	0.02 ± 0.01	26.43 ± 1.43	2.23 ± 1.52	0.66 ± 0.12	6.32 ± 0.32	4.12 ± 0.01

The values presented are mean ± S.E.M (n = 8); ** *p* < 0.01 and * *p* < 0.05 vs. OVX, ^#^ *p* < 0.05, ^##^ *p* < 0.01, vs. sham. SHAM: sham (control), OVX: ovariectomized control, RLX: raloxifene hydrochloride, NRG: naringenin, NRG-NS: naringenin nanosuspension.

## Data Availability

Data contained within the article; additional data available from S.G., S.S. on reasonable request, and S.G., S.S. are responsible for the data provided in the manuscript.
